# The complete chloroplast genome sequence of *Centaurea cyanus* (Asteraceae)

**DOI:** 10.1080/23802359.2023.2185470

**Published:** 2023-03-13

**Authors:** NingYun Zhang, Peng Xie, Kerui Huang, Hanbin Yin, Ping Mo, Yun Wang

**Affiliations:** Hunan Provincial Key Laboratory for Molecular Immunity Technology of Aquatic Animal Diseases, College of life and environmental sciences, Hunan University of Arts and Science, Hunan, China

**Keywords:** Centaurea cyanus, phylogenetic analysis, chloroplast genome

## Abstract

*Centaurea cyanus* has been a weed in farmland for a long time. In this study, the chloroplast genome of *C. cyanus* was sequenced to establish the phylogenetic relationship between its genomic characteristics and other related species. The chloroplast gene structure of *C. cyanus* is a circular molecule with a length of 152,433 bp, including a large single-copy (LSC) region of 83,464 bp, a small single-copy (SSC) region of 18,545 bp, and a pair of inverted repeats sequences (IRs) region of 25,212 bp. The whole genome contains 130 genes, including 86 protein-coding genes, 36 tRNA genes, and eight rRNA genes. Phylogenetic analysis showed that *C. cyanus* is close to *Carthamus. tinctorius*, *C. tinctorius, C. diffusa,* and *C. maculosa,* and all of them were in one clade. This study provides genetic resource information for the further study of *Centaurea*.

## Introduction

**Figure 1.**
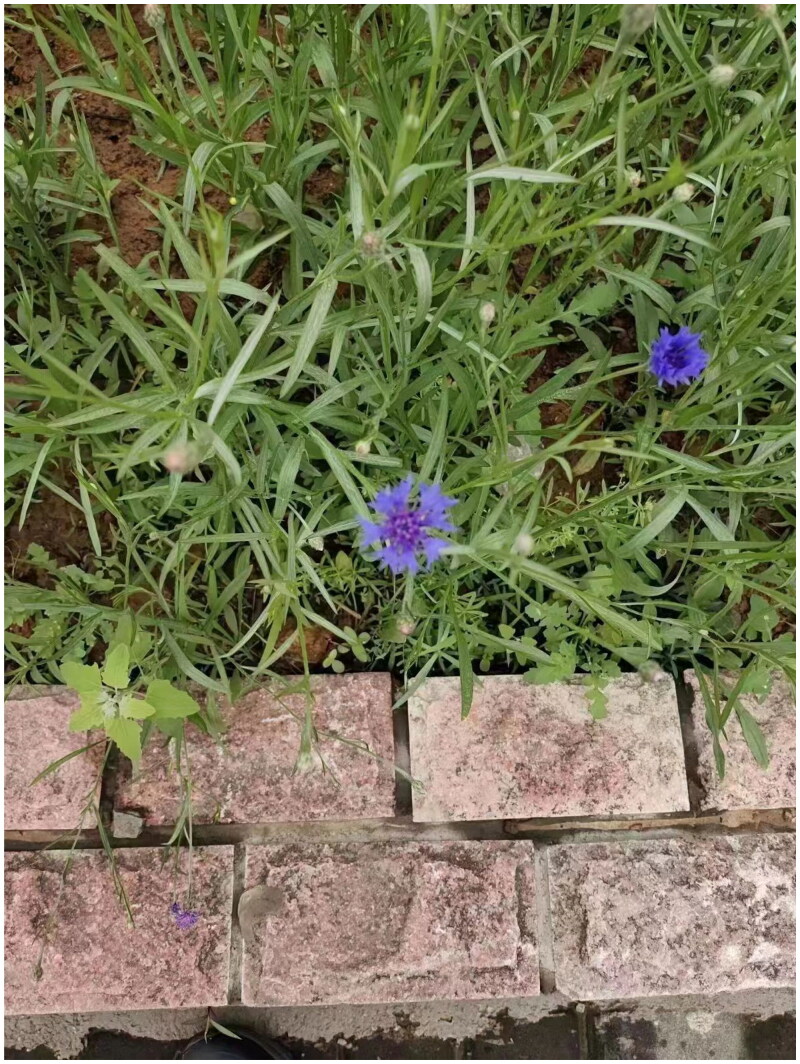
The picture of the collected sample of *Centaurea cyanus. Note*. The picture is self-taken nearby the Changde Vocational Technical College, Changde, Hunan province, China (N29°02′29.76″, E111°38′05.32″, 34 m).

*Centaurea cyanus* Linnaeus 1753 also known as ‘blue cornflower’ or ‘bachelor’s button’, belongs to the family Asteraceae (Figure 1). *C. cyanus* is annual or biennial, with a height of 30–70 cm or higher. It has grey-green branched stems and a tap root system with lanceolate leaves alternately arranged on the stem 1–4 cm long. Its flowerheads are 1.5–3 cm in diameter, with capitula structured with deep blue sterile ray florets and less showy fertile disk florets, containing a single ovule in each ovary (Tomar 2017; Haratym et al. 2020). It is native to Europe, and was also introduced to North America, where it is considered an invasive species. It appears in autumn and spring fields and often infects winter crops. It has been a weed in farmland for a long time, mainly growing in corn fields or along the edge of farmland (Haratym et al. 2020). Although it is a weed, *C. cyanus* is a popular garden plant for its unique blue color. In addition, *C. cyanus* is widely used in the medical field. Its flowers are used as diuretics in the Russian Federation’s current pharmacopoeia (Shikov et al. 2017) and also as diuretics and supplements in Scottish medicine (Bouafia et al. 2020). Its flowers are used in European phytotherapy for treating minor ocular inflammation (Garbacki et al. 1999). The polysaccharides extracted from its flower heads had anti-inflammatory properties, which was shown in a previous pharmacological experiment (Pirvu et al. 2012). There are many terpenes belonging to sesquiterpenes making up the main compound of the essential oil extracted from the aerial parts of *C. 9cyanus*, they have the potential to treat cancer and cardiovascular diseases and also have the effects of preventing neurodegeneration and treating burns, thus they are used as pharmaceutical preparations (Chadwick et al. 2013). *C. cyanus* is also of great significance in the cosmetics industry. Aromatic acids extracted from *C. cyanus* are one of the most common raw materials in cosmetics production (Pirvu et al. 2012). In addition, *C. cyanus* has many other functions, which will not be described here. Although *C. cyanus* has been utilized in many fields, its phylogeny has not been fully resolved, and its chloroplast genome structure and feature are still unknown.

In this study, the chloroplast genome was sequenced and characterized to explore its phylogenetic status, and phylogenetic analysis was performed to provide information for further phylogenetic studies.

## Materials

For plant materials, fresh leaves were picked from *Centaurea cyanus* cultivated near the Changde Vocational Technical College, Changde, Hunan province, China (N29°02′29.76″, E111°38′05.32″, Altitude: 34 m). The voucher specimen was preserved at the College of Life and Environmental Sciences, Hunan University of Arts and Sciences (Contact Person: Kerui Huang, huangkerui008@163.com, voucher number SCJ003).

## Methods

Total DNA was extracted from the leaves stored in liquid nitrogen using a DN easy plant tissue kit (TIANGEN Biotech Co., Ltd., Beijing). Then the library was constructed and sequenced using Illumina HiSeq 2500 platform (Shanghai personalbio Technology Co., Ltd., China). As a result, 67,498,968 reads were retained after filtering out the low-quality reads using fastp (Chen et al. 2018). The de novo assembly of the *C. cyanus* chloroplast genome was performed using GetOrganelle v1.7.5 (Jin et al. 2020), of which the detailed information for assembling is shown in Figure S1, and the CPGAVAS2 (Shi et al. 2019) was used for the chloroplast genome annotation. Finally, the genome map was drawn using CPGView (Liu et al. 2023). Phylogenetic analysis was performed through the following procedure: Firstly, a total of 51 chloroplast genomes were downloaded from GenBank, 73 protein-coding genes shared by all genomes were screened, and after that, MAFFT v7.313 (Rozewicki et al. 2019) was used for separate alignment of each gene. Then Gblocks 0.91b was used for sequence masking of each gene, and end-to-end connections of all the genes were performed to form a supergene of each species (Guo et al. 2022). Maximum likelihood phylogenies were inferred using IQ-TREE (Nguyen et al. 2015) under the TVM + F + I + G4 model for 5000 ultrafast bootstraps, as well as the Shimodaira–Hasegawa–like approximate likelihood-ratio test.

## Results

**Figure 2.**
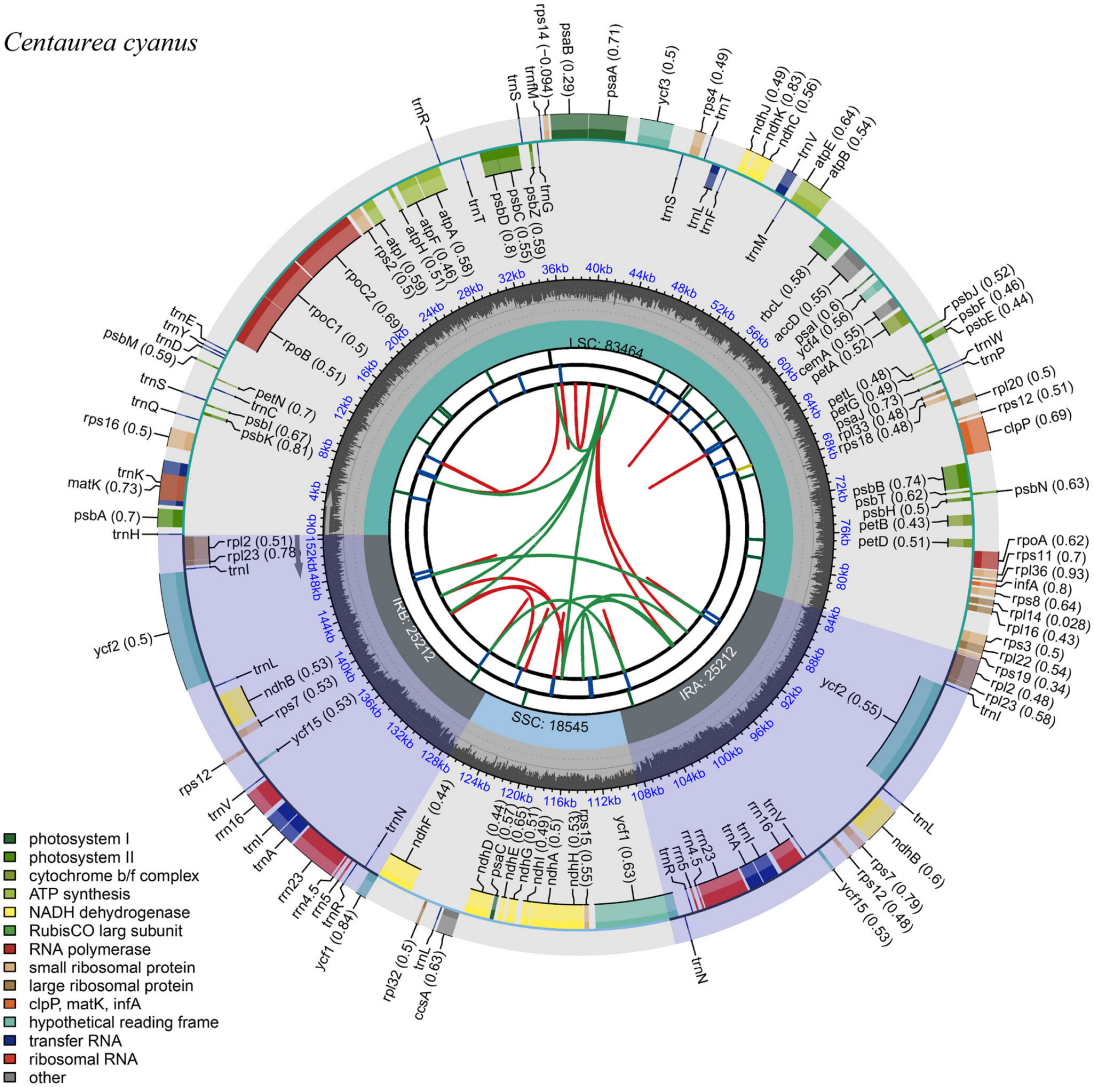
Gene map of the *Centaurea cyanus* chloroplast genome. From the center outward, the first track indicates the dispersed repeats; The second track shows the long tandem repeats as short blue bars; The third track shows the short tandem repeats or microsatellite sequences as short bars with different colors; The fourth track shows small single-copy (SSC), inverted repeat (Ira and Irb), and large single-copy (LSC) regions. The GC content along the genome is plotted on the fifth track; The genes are shown on the sixth track.

**Figure 3.**
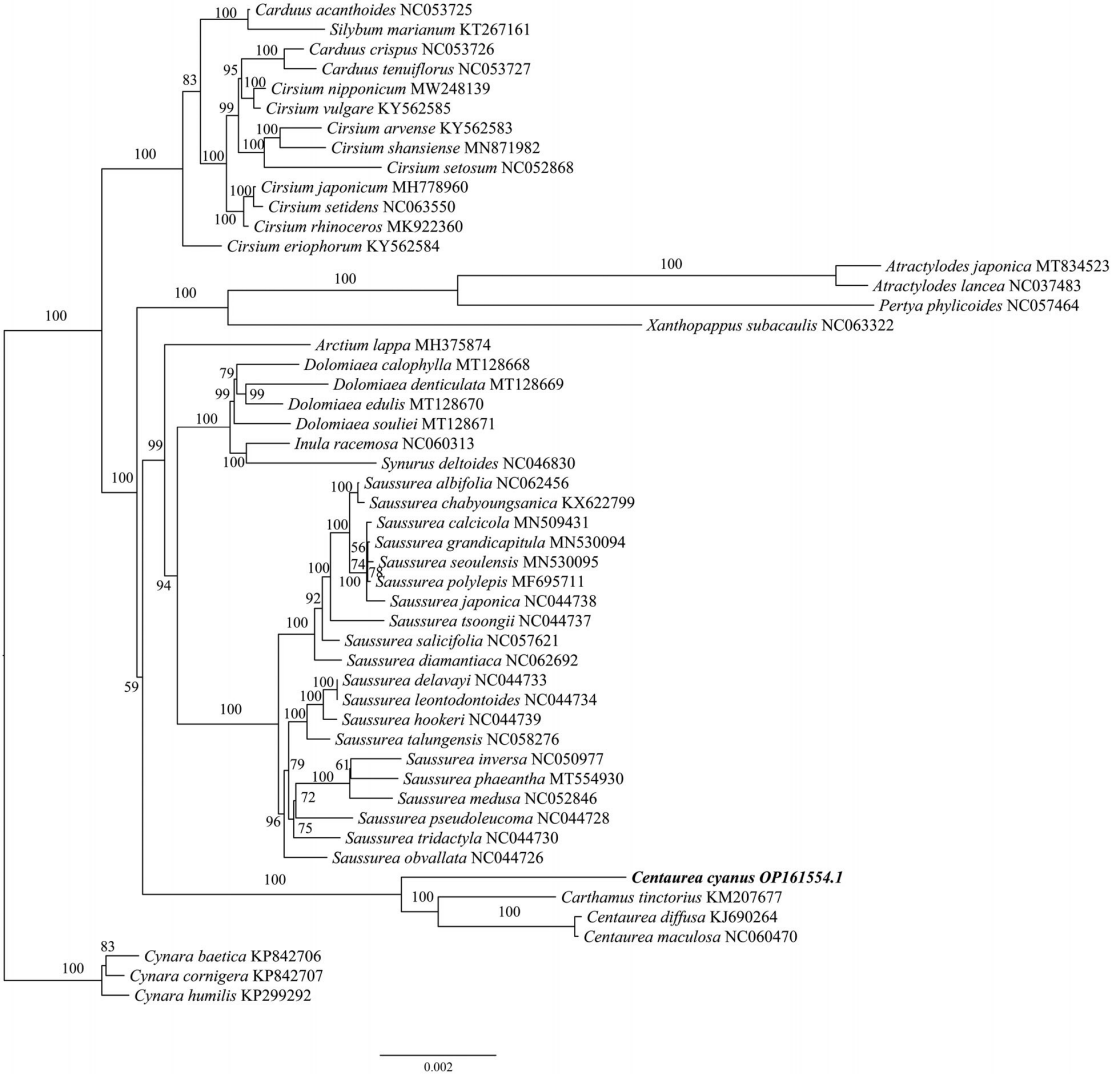
Maximum-likelihood (ML) tree of *Centaurea cyanus* and 49 relative species was reconstructed using the IQ-Tree based on 73 protein-coding genes shared by all genomes. Bootstrap values are shown next to the nodes. The following sequences, of which some existed in NCBI database but were unpublished, were used: *Carduus acanthoides* NC053725, *Silybum marianum* KT267161, *Carduus crispus* NC053726, *Carduus tenuiflorus* NC053727, *Cirsium nipponicum* MW248139, *Cirsium vulgare* KY562585, *Cirsium arvense* KY562583 (Jung et al. 2021), *Cirsium shansiense* MN871982 (Xu et al. 2020), *Cirsium setosum* NC052868, *Cirsium japonicum* MH778960 (Yu and Kim 2019), *Cirsium setidens* NC063550, *Cirsium rhinoceros* MK922360 (Nam et al. 2019), *Cirsium eriophorum* KY562584, *Atractylodes japonica* MT834523 (Shi et al. 2021), *Atractylodes lancea* NC037483, *Pertya phylicoides* NC057464, *Xanthopappus subacaulis* NC063322, *Arctium lappa* MH375874 (Xing et al. 2019), *Dolomiaea calophylla* MT128668, *Dolomiaea denticulata* MT128669, *Dolomiaea edulis* MT128670, *Dolomiaea souliei* MT128671, *Inula racemosa* NC060313, *Synurus deltoides* NC046830, *Saussurea albifolia* NC062456, *Saussurea chabyoungsanica* KX622799 (Cheon et al. 2017), *Saussurea calcicola* MN509431, *Saussurea grandicapitula* MN530094, *Saussurea seoulensis* MN530095, *Saussurea polylepis* MF695711 (Yun et al. 2017), *Saussurea japonica* NC044738, *Saussurea tsoongii* NC044737, *Saussurea salicifolia* NC057621, *Saussurea diamantiaca* NC062692, *Saussurea delavayi* NC044733, *Saussurea leontodontoides* NC044734, *Saussurea hookeri* NC044739, *Saussurea talungensis* NC058276, *Saussurea inversa* NC050977, *Saussurea phaeantha* MT554930, *Saussurea medusa* NC052846, *Saussurea pseudoleucoma NC044728*, *Saussurea tridactyla* NC044730, *Saussurea obvallata* NC044726, *Carthamus tinctorius* KM207677, *Centaurea diffusa KJ690264*, *Centaurea maculosa* NC060470, *Cynara baetica KP842706*, *Cynara cornigera* KP842707, *Cynara humilis* KP299292 (Curci and Sonnante 2016).

The chloroplast gene structure of *Centaurea cyanus* is a circular molecule (Figure 2), with a length of 152,433 bp, including four parts: a large single-copy region (LSC) length of 83,464 bp, a small single-copy region (SSC) length of 18,545 bp, and two inverted repeat regions (IRs), each 25,212 bp. The G + C content was 37.76% for the whole chloroplast genome, and 43.14% for the IRs, which was higher than that in LSC and SSC regions (35.93% and 31.38%, respectively). The genome contains 130 genes, including 86 protein-coding genes, 36 tRNA genes, and eight rRNA genes, and the structure of the cis-splicing genes and trans-splicing genes were shown in supplemental Figure S2. Based on the chloroplast genome of *C. cyanus,* the Maximum-likelihood (ML) tree was constructed (Figure 3), which shows the phylogenetic placement of *Centaurea cyanus*. The result showed that *C. cyanus* is close to, *Carthamnus tinctorius*, *C. diffusa*, and *C. maculosa*, and all of them were in one clade with high support, which is consistent with the previous study (Garcia-Jacas et al. 2001), however, *Carthamus tininctorius* is also presented in the clade of *Centaurea*, which requires further study.

## Discussion and conclusion

The study reports the chloroplast genome of *Centaurea. cyanus* for the first time, the phylogenetic result is basically consistent with the previous study (Garcia-Jacas et al. 2001). However, *Carthamus tininctorius* is also presented in the clade of *Centaurea*, which is an interesting phenomenon because the circumscription of Centaurea is still not clear yet (Garcia-Jacas et al. 2001). Besides, only a few chloroplast genomes of the genus *Centaurea* were reported. Thus, further research is needed to understand the phylogenetic location of *Centaurea*. This study provides genetic resource information for the further study of *Centaurea*.

## Supplementary Material

Supplemental Material

## Data Availability

The complete chloroplast genome sequence of *Centaurea cyanus* has been deposited in the GenBank database under the accession number NC066898 or OP161554 (these numbers were automatically generated by NCBI and refer to the same sample). The associated BioProject, SRA, and Bio-Sample numbers are PRJNA891141, SRR21929279, and SAMN31311817, respectively.

## References

[CIT0001] Bouafia M, Benarfa A, Gourine N, Yousfi M. 2020. Seasonal variation of fatty acid composition, tocopherol content and antioxidant activity of lipid extracts from *Centaurea* sp. Food Biosci. 37:100728.

[CIT0002] Chadwick M, Trewin H, Gawthrop F, Wagstaff C. 2013. Sesquiterpenoids lactones: benefits to plants and people. Int J Mol Sci. 14(6):12780–12805. 23783276 10.3390/ijms140612780 PMC3709812

[CIT0003] Chen S, Zhou Y, Chen Y, Gu J. 2018. fastp: an ultra-fast all-in-one FASTQ preprocessor. Bioinformatics. 34(17):i884–i890. 30423086 10.1093/bioinformatics/bty560 PMC6129281

[CIT0004] Cheon KS, Kim HJ, Han JS, Kim KA, Yoo KO. 2017. The complete chloroplast genome sequence of *Saussurea chabyoungsanica* (Asteraceae), an endemic to Korea. Conserv Genet Resour. 9(1):51–53.

[CIT0005] Curci PL, Sonnante G. 2016. The complete chloroplast genome of *Cynara humilis*. Mitochondrial DNA A DNA Mapp Seq Anal. 27(4):2345–2346. 25812057 10.3109/19401736.2015.1025257

[CIT0006] Garbacki N, Gloaguen V, Damas J, Bodart P, Tits M, Angenot L. 1999. Anti-inflammatory and immunological effects of Centaurea cyanus flower-heads. J Ethnopharmacol. 68(1–3):235–241. 10624883 10.1016/s0378-8741(99)00112-9

[CIT0007] Garcia-Jacas N, Susanna A, Garnatje T, Vilatersana R. 2001. Generic delimitation and phylogeny of the subtribe Centaureinae (Asteraceae): a combined nuclear and chloroplast DNA analysis. Ann Bot. 87(4):503–515.

[CIT0008] Guo S, Liao XJ, Chen SY, Liao BS, Guo YM, Cheng RY, Xiao SM, Hu HY, Chen J, Pei J, et al. 2022. A Comparative analysis of the chloroplast genomes of fourpolygonum medicinal plants. Front Genet. 13:764534. 35547259 10.3389/fgene.2022.764534 PMC9084321

[CIT0009] Haratym W, Weryszko-Chmielewska E, Konarska A. 2020. Microstructural and histochemical analysis of aboveground organs of *Centaurea cyanus* used in herbal medicine. Protoplasma. 257(1):285–298. 31515607 10.1007/s00709-019-01437-4 PMC6982636

[CIT0010] Jin J-J, Yu W-B, Yang J-B, Song Y, dePamphilis CW, Yi T-S, Li D-Z. 2020. GetOrganelle: a fast and versatile toolkit for accurate de novo assembly of organelle genomes. Genome Biol. 21(1):241. 32912315 10.1186/s13059-020-02154-5 PMC7488116

[CIT0011] Jung J, Do HD, Hyun J, Kim C, Kim JH. 2021. Comparative analysis and implications of the chloroplast genomes of three thistles (Carduus L, Asteraceae). PeerJ. 9:e10687. 33520461 10.7717/peerj.10687 PMC7811785

[CIT0012] Liu S, Ni Y, Li J, Zhang X, Yang H, Chen H, Liu C. 2023. CPGView: a package for visualizing detailed chloroplast genome structures. Mol Ecol Resour. 0:1–11. 10.1111/1755-0998.13729 36587992

[CIT0013] Nam S, Kim J, Kim Y, Ku J, Jung S, Lee Y, Kim S, Xi H, Song J, Park J. 2019. The complete chloroplast genome of Korean endemic species, *Cirsium rhinoceros* (H.Lév. & vaniot) Nakai (Asteraceae). Mitochondrial DNA B Resour. 4(2):2351–2352. 33365539 10.1080/23802359.2019.1627940 PMC7687605

[CIT0014] Nguyen LT, Schmidt HA, von Haeseler A, Quang Minh B. 2015. IQ-TREE: a fast and effective stochastic algorithm for estimating maximum-likelihood phylogenies. Mol. Biol. Evol. 32(1):268–274. 25371430 10.1093/molbev/msu300 PMC4271533

[CIT0015] Pirvu L, Dragomir C, Schiopu S, Colceru Mihul S. 2012. Vegetal extracts with gastroprotective activity. Part. I. Extracts obtained from *Centaurea cyanus* L. raw material. Rom Biotechnol Lett. 17(2):7169–7176.

[CIT0016] Rozewicki J, Li S, Mar Amada K, M Standley D, Katoh K. 2019. MAFFT-DASH: integrated protein sequence and structural alignment. Nucleic Acids Res. 47(W1):W5–W10. 31062021 10.1093/nar/gkz342 PMC6602451

[CIT0017] Shi LC, Chen HM, Jiang M, Wang L, Wu X, Huang LF, Liu C. 2019. CPGAVAS2, an integrated plastome sequence annotator and analyzer. Nucleic Acids Res. 47(W1):W65–W73. 31066451 10.1093/nar/gkz345 PMC6602467

[CIT0018] Shi M, Xie H, Zhao C, Shi L, Liu J, Li Z. 2021. The complete chloroplast genome of *Atractylodes japonica* Koidz. ex Kitam. and its phylogenetic inference. Mitochondrial DNA B Resour. 6(7):2038–2040. 34377788 10.1080/23802359.2021.1927217 PMC8344787

[CIT0019] Shikov A, Tsitsilin A, Pozharitskaya O, Makarov VG, Heinrich M. 2017. Traditional and current food use of wild plants listed in the Russian pharmacopoeia. Front Pharmacol. 8:841. 29209213 10.3389/fphar.2017.00841 PMC5702350

[CIT0020] Tomar A. 2017. Medicinal use of *Centaurea cyanus* Linn. to cure ophthalmia. J Pharm Phytochem. 6(5):232–233.

[CIT0021] Xing YP, Xu L, Chen SY, Liang YM, Wang JH, Liu CS, Kang TG. 2019. Comparative analysis of complete chloroplast genomes sequences of *Arctium lappa* and *A. tomentosum*. Biol Plantarum. 63:565–574.

[CIT0022] Xu J, Dang H, Li H. 2020. The complete chloroplast genome of *Cirsium shansiense* and its phylogenetic analysis. Mitochondrial DNA Part B. 5(2):1134–1135.

[CIT0023] Yu GE, Kim CK. 2019. The complete chloroplast genome of *Cirsium japonicum* (Asterales: Asteraceae). Mitochondrial DNA Part B. 4(1):1812–1813.

[CIT0024] Yun SA, Gil HY, Kim SC. 2017. The complete chloroplast genome sequence of *Saussurea polylepis* (Asteraceae), a vulnerable endemic species of Korea. Mitochondrial DNA B Resour. 2(2):650–651. 33473934 10.1080/23802359.2017.1375881 PMC7799718

