# Supplementary material for: The complete chloroplast genome sequence of Centaurea cyanus (Asteraceae)
Source: Mitochondrial DNA B Resour. 2023 Mar 13;8(3):393–7. doi: 10.1080/23802359.2023.2185470 (PMC10013558; doi:10.1080/23802359.2023.2185470)
Supplement: Supplemental Material [file TMDN_A_2185470_SM2729.docx]

**Supplemental Material**


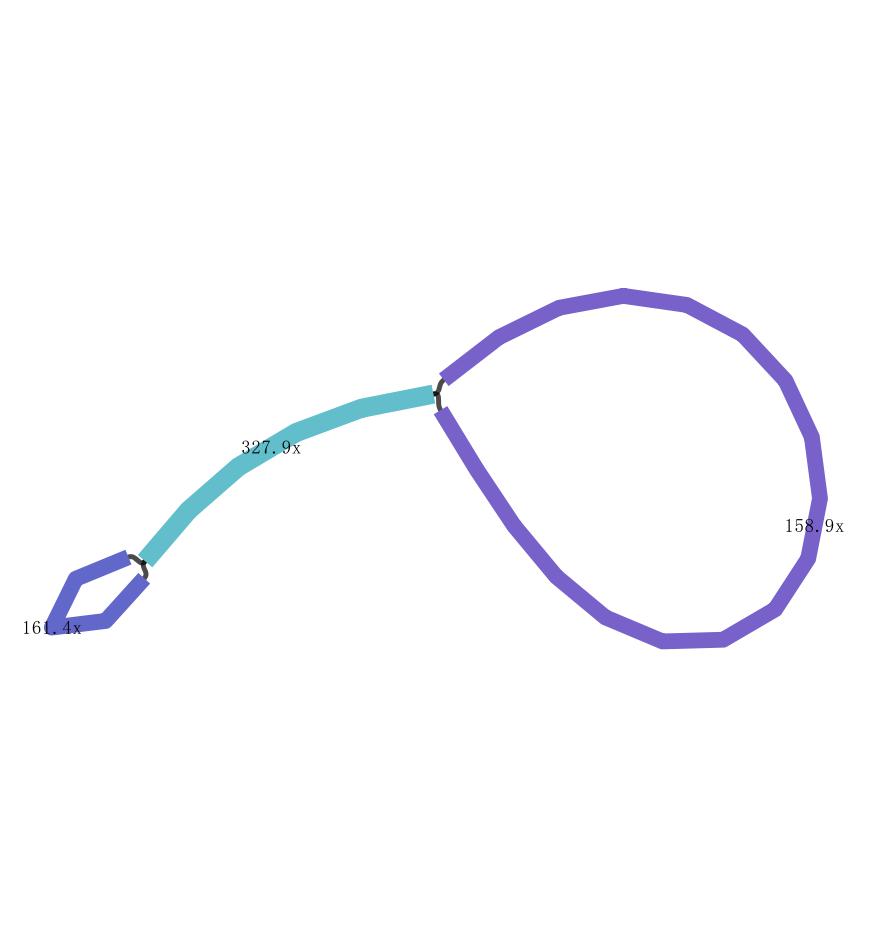


Figure S1. The schematic representation of the coverage depth for the entire chloroplast genome of *Centaurea cyanus* using Bandage. The numbers indicate the depths of different regions.


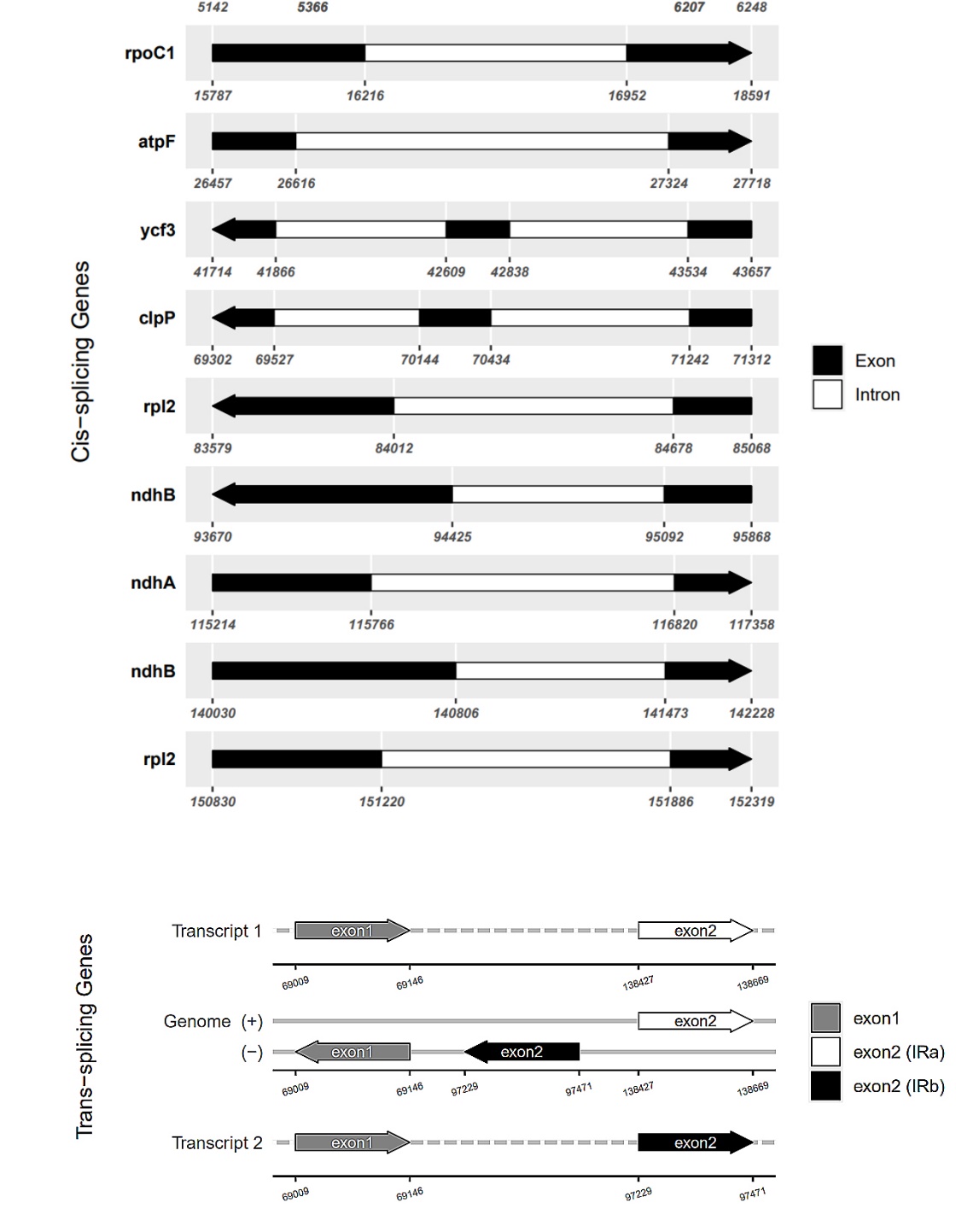


Figure S2. Schematic map of the cis-splicing genes and trans-splicing gene rps12 in the chloroplast genome of *Centaurea cyanus* using CPGView. The exons of the cis-splicing genes are shown in black; the introns are shown in white. The arrow indicates the sense direction of the gene. Please note that lengths of exons and introns are not drawn to scale.
